# Nature of the evidence base and approaches to guide nutrition interventions for individuals: a position paper from the Academy of Nutrition Sciences

**DOI:** 10.1017/S0007114524000291

**Published:** 2024-05-28

**Authors:** Mary Hickson, Constantina Papoutsakis, Angela M Madden, Mary Anne Smith, Kevin Whelan

**Affiliations:** 1 University of Plymouth, Plymouth, PL4 6AB Devon, UK; 2 British Dietetic Association, Birmingham, UK; 3 Academy of Nutrition and Dietetics, Nutrition and Dietetics Data Science Centre, Research, International, and Scientific Affairs (RISA), Chicago, USA; 4 University of Hertfordshire, Hatfield, UK; 5 Dietitians of Canada, Toronto, Canada; 6 King’s College London, Department of Nutritional Sciences, London, UK; 7 Academy of Nutrition Sciences, London, UK

**Keywords:** Consensus recommendations, Academy Nutritional Sciences, Individualised, Nutrition Interventions, Evidence Base

## Abstract

This Position Paper from the Academy of Nutrition Sciences is the third in a series which describe the nature of the scientific evidence and frameworks that underpin nutrition recommendations for health. This paper focuses on evidence which guides the application of dietary recommendations for individuals. In some situations, modified nutrient intake becomes essential to prevent deficiency, optimise development and health, or manage symptoms and disease progression. Disease and its treatment can also affect taste, appetite and ability to access and prepare foods, with associated financial impacts. Therefore, the practice of nutrition and dietetics must integrate and apply the sciences of food, nutrition, biology, physiology, behaviour, management, communication and society to achieve and maintain human health. Thus, there is huge complexity in delivering evidence-based nutrition interventions to individuals. This paper examines available frameworks for appraising the quality and certainty of nutrition research evidence, the development nutrition practice guidelines to support evidence implementation in practice and the influence of other sources of nutrition information and misinformation. The paper also considers major challenges in applying research evidence to an individual and suggests consensus recommendations to begin to address these challenges in the future. Our recommendations target three groups; those who deliver nutrition interventions to individuals, those funding, commissioning or undertaking research aimed at delivering evidence-based nutrition practice, and those disseminating nutritional information to individuals.

## Graphical abstract

**Figure:**
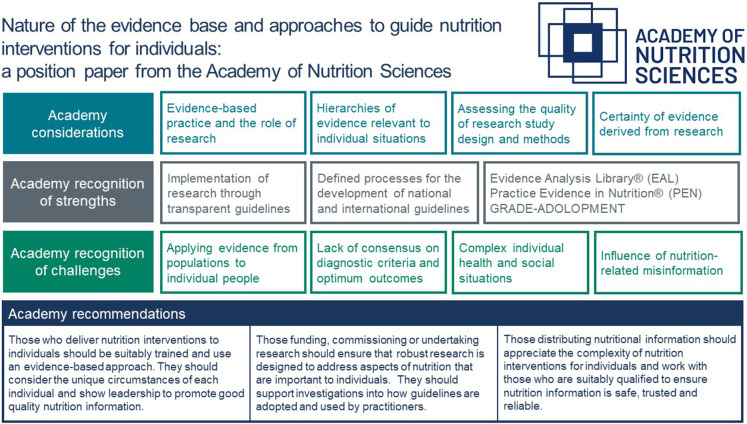

Diet is key to the maintenance of health and crucial in the prevention and management of many diseases. Thus, it is important that dietary interventions are based on sound evidence and that nutrition professionals apply this evidence when working with individuals. This third position paper from the Academy of Nutrition Sciences (ANS) examines how evidence is used to guide individualised nutrition interventions. It builds upon the first ANS position paper^(1)^ that focussed on how dietary recommendations are formulated for populations for prevention of non-communicable diseases and the second ANS position paper^(2)^, that examined the evidence used to support health claims for specific foods.

Individual requirements for energy, macronutrients and micronutrients are deeply impacted by factors such as life stage (growth, pregnancy etc.) and health status, which can affect the process of consuming, digesting, absorbing, metabolising or excreting nutrients. In some situations, modified nutrient intake becomes essential to prevent deficiency, optimise development and health, or manage symptoms and disease progression. Disease and its treatment can also affect taste, appetite and ability to access and prepare foods, with associated financial impacts. Therefore, the practice of nutrition and dietetics must integrate and apply the sciences of food, nutrition, biology, physiology, behaviour, management, communication and society to achieve and maintain human health. Thus, there is huge complexity in delivering evidence-based nutrition interventions to individuals.

A primary way in which evidence is used to guide individualised nutrition interventions is through the development of clinical practice guidelines, which are ‘systematically developed statements to assist practitioner and patient decisions about appropriate health care for specific clinical circumstances’^(3)^. Guidelines attempt to bridge the gap between research and clinical practice, guiding the practitioner and patient to implement treatments based on the best available evidence. In this position paper, we specifically discuss nutrition practice guidelines – guidelines focussed on nutritional care – rather than guidelines that include nutrition among other interventions. Nutrition practice guidelines should make the task of implementing evidence-based individualised nutrition interventions easier for the practitioner.

The aim of this ANS position paper is to provide a state-of-the-art summary of how evidence-based practice, with a particular emphasis on research evaluation, is used to inform nutrition interventions for individuals. The paper also considers major challenges in applying research evidence to an individual and suggests consensus recommendations to begin to address these challenges for the future. Our recommendations target three groups; those who deliver nutrition interventions to individuals, those funding, commissioning or undertaking research aimed at delivering evidence-based nutrition practice and those disseminating nutritional information to individuals.

## Evidence-based practice and the role of research

In this section, we first define evidence-based practice before delving into a deeper description of research, critical appraisal and challenges applying nutrition research in practice.

Research can be defined as ‘the attempt to derive generalisable or transferable new knowledge to answer or refine relevant questions with scientifically sound methods’^(4)^ (See Table 1). It is important to distinguish between research and ‘audit’ (review of care against explicit criteria and the implementation of change) and ‘service evaluation’ (to describe, measure or judge current care) (See Table 1 Glossary). Although audit and service evaluation are important quality assurance and quality improvement approaches, they do not fulfil the definition of research and cannot be used to produce reliable evidence for the efficacy of an intervention.

**Table 1.** Glossary of common terms in relation to research, evidence-based practice and clinical guidelines

Term	Definition
Anecdotal evidence	Observations made through clinical practice which link a particular outcome with a particular condition or intervention.
Audit	A quality improvement process that reviews the structure, processes and outcomes of care which are systematically evaluated against explicit criteria, followed by the implementation of change (at an individual, team or service level) and further monitoring to confirm improvement in patient care and outcomes.
Case-control study	People with a particular condition (cases) are matched with people without the condition (controls), and differences are then examined in a retrospective analysis.
Case report or case series	A report based on a single patient which can sometimes be collected into a short series.
Clinical guidelines	Clinical guidelines are recommendations on how healthcare and other professionals should care for people with specific conditions. The recommendations are based on the best available evidence.
Cohort study	Two or more groups of people who have a different exposure to a particular agent (like type of diet, nutrient, smoking, a vaccine, etc) are selected and then followed up to see what differences there are between the groups for specific outcomes.
Cross-sectional studies and surveys	An examination of a sample of the population of interest at one point in time. Surveys often use a questionnaire or interview design.
Domiciled feeding study	Involve subjects being housed and fed in comfortable yet controlled facilities.
Efficacy	The performance of an intervention under controlled, ideal circumstances.
Effectiveness	The performance of an intervention in ‘real-world’ conditions, considering acceptability, adherence and feasibility.
Evidence-based nutrition practice	Using the best available nutrition evidence, together with clinical experience, to help patients prevent (sometimes), resolve (sometimes) or cope with (often) problems related to their physical, mental and social health, according to their values and preferences.
Evidence-based practice	The conscientious, explicit and judicious use of current best evidence in making decisions about the care of individual patients.
Expert opinion	A consensus from the experts in the field.
Generalisability	The extent to which the findings of a study can be applied to other situations.
Health research	Any research into matters relating to people’s physical or mental health. Excludes anything involving animal research.
Interventional research	Research involving a change in treatment, care or other services made for the purpose of the research.
Meta-analysis	A meta-analysis is the statistical combination of results from several independent trials to produce an overall result. A systematic review will often include a meta-analysis.
N-of-1 trial	Single participant study whereby a single patient is the entire trial, a single case study. A trial in which random allocation can be used to determine the order in which an experimental and a control intervention are given to a patient is an N-of-1 randomised controlled trial. The order of experimental and control interventions can also be fixed.
Qualitative research	Qualitative research involves the analysis of words, pictures or objects in order to explore and describe a situation, process or experience. The researcher interprets the data as it unfolds during the collection process and the subjective nature of the process is embraced.
Quality Improvement	Quality improvement is the series of activities used in healthcare to systematically improve care. Quality improvement seeks to standardise processes and structure to reduce variation, achieve predictable results and improve outcomes for patients, healthcare systems and organisations. Structure includes technology, culture, leadership and physical capital; process includes knowledge capital (e.g. standard operating procedures) or human capital (e.g. education and training).
Quantitative research	Quantitative research involves the analysis of numerical data to explain a pre-defined hypothesis. The researcher aims to remain objective and control the conditions of the research.
Randomised controlled trial	Trial participants are randomly assigned to either a control group or a treatment group who receive a specific intervention. The aim is to control all other factors that may influence the effect of the intervention.
Research	The attempt to derive generalisable or transferable new knowledge to answer or refine relevant questions with scientifically sound methods.
Risk of Bias	This is the likelihood that features of the study design or conduct of the study will give misleading results.
Service evaluation	Designed and conducted solely to define, measure or judge current care. It measures current service without reference to a standard or explicit criteria (unlike audit) and does not involve an intervention (any choice of treatment is in line with routine clinical practice).
Social care research	Any research into matters relating to personal care or other practical assistance for individuals who are in need of care or assistance because of age, physical or mental illness, disability, pregnancy, childbirth, dependence or other similar circumstances.
Systematic review	A systematic review combines the results of a number of original studies which have been systematically searched out, judged on pre-defined inclusion criteria, and a synthesis of their results is produced.
Transferability	Transferability is similar to generalisability or external validity in quantitative research. It measures whether, or to what extent, the study’s results are applicable within other contexts, circumstances and settings.
Internal Validity (of diagnostic criteria)	Internal validity of diagnostic criteria shows whether the components that contribute to the diagnosis are reflected in the diagnostic outcome. This validity is influenced by the methods used to reduce the risk of bias and chance when developing the criteria
External Validity (of diagnostic criteria)	External validity of diagnostic criteria considers the usefulness and applicability of the criteria and examines how well the criteria compare to other measures for diagnosing the same condition
Prognostic Validity (of diagnostic criteria)	Prognostic validity considers how well the criteria are associated with clinical outcomes, that is do they predict health risk or benefit in the future

Adapted from^(5–8)^.

**Fig. 1.**
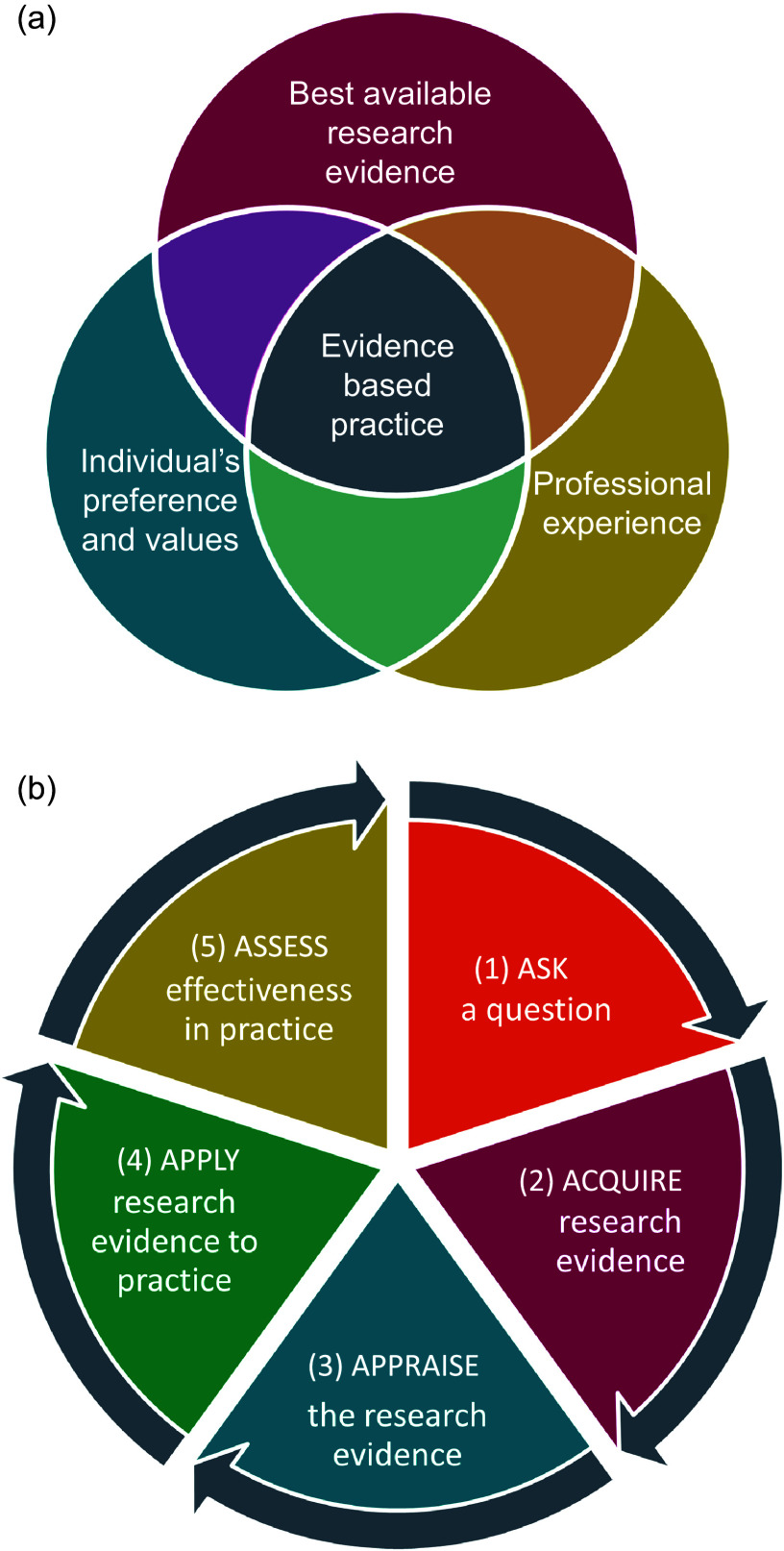
Models of evidence-based practice. (a) Evidence-based practice integrates individual clinical expertise with the best available external clinical evidence from systematic research and the patient’s preferences. (b) Evidence-based practice can be achieved through a 5-step model (5As): ask a question; acquire the evidence; appraise the evidence; apply the evidence; and assess the effectiveness.

Evidence-based practice (originally termed ‘evidence-based medicine’) can be defined as the ‘conscientious, explicit and judicious use of current best evidence in making decisions about the care of individual patients’^(5)^. Importantly, evidence-based practice recognises that research evidence is integrated with clinical expertise and patient preference (Fig. 1). This has been referred to as a ‘three-legged stool’ to emphasise that without one of these elements, evidence-based clinical decision-making collapses^(9)^. The integration of all three elements leads to potential improvements in outcome or reductions in harm and therefore may improve both effectiveness and patient safety, whilst also considering the acceptability of an intervention and therefore likely uptake and adherence. This makes the application of research evidence in delivery of nutrition and dietary interventions an *art* as well as a *science*. The term ‘evidence-based nutrition practice’ has been coined to acknowledge the specific issues related to nutrition and dietetics, which has been defined as ‘using the best available nutrition evidence, together with clinical experience, to help patients prevent (sometimes), resolve (sometimes) or cope with (often) problems related to their physical, mental and social health, according to their values and preferences’^(6)^. As such, evidence-based nutrition practice involves three fundamental principles, summarised in Box 1.

Box 1 Common principles of evidence-based practice specific to nutrition^(6)^.

- Optimal clinical decision-making requires awareness of the best available evidence that will ideally come from systematic summaries of the available evidence.
- Evidence-based nutrition provides guidance to decide whether evidence is more or less trustworthy, that is, how certain can we be of patients’ prognosis, the properties of diagnostic tests and of the therapeutic options?
- Evidence alone is insufficient to make a clinical decision. Practitioners of evidence-based nutrition must always trade off the benefits with the risks, burden and costs associated with alternative management strategies and, in so doing, consider patients’ unique predicament, including their values and preferences.

### Frameworks for appraising the quality and certainty of research evidence

Central to evidence-based practice and evidence-based nutrition practice is the ability to appraise research. Comprehensive critical appraisal should assess three components: (1) whether an appropriate study design has been used to answer the clinical question; (2) the methodological quality of the study (i.e. specific aspects of the methods); and (3) the certainty of the evidence by applying GRADE or similar method.

#### Assessing the quality of study design: hierarchies of evidence

**Fig. 2.**
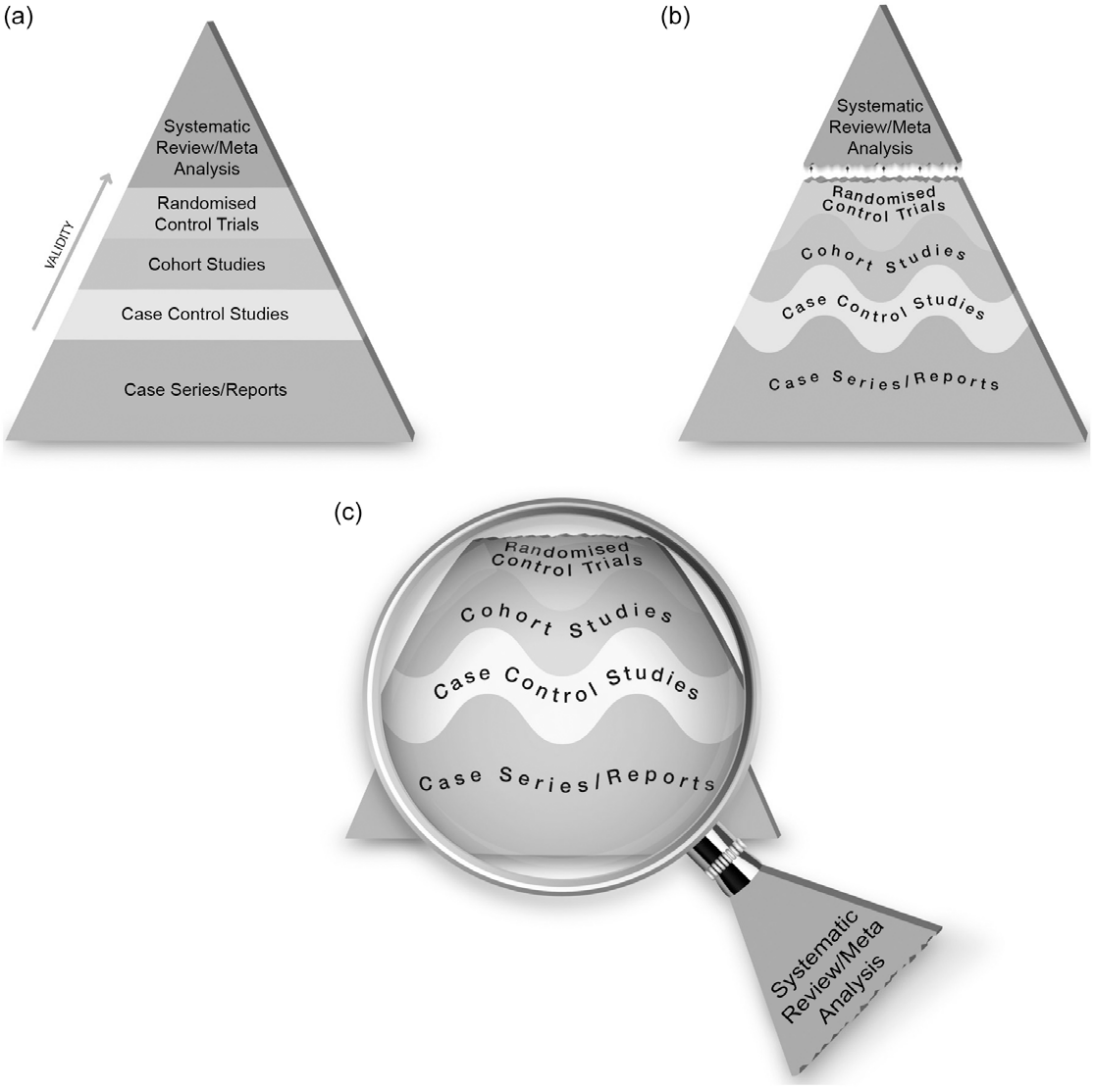
Traditional and proposed alterations to hierarchies of evidence to support the efficacy of an intervention. (a) The traditional pyramid ranging from case reports and case series at the bottom and systematic reviews and meta-analyses at the top; (b) lines separating the study designs become wavy as a result of variations in study quality, and systematic reviews are separated from the pyramid; (c) lines separating the study designs become wavy as a result of variations in study quality, and systematic reviews are no longer at the top of the hierarchy but instead a lens through which evidence is viewed. Taken with permission from^(14)^.

**Fig. 3.**
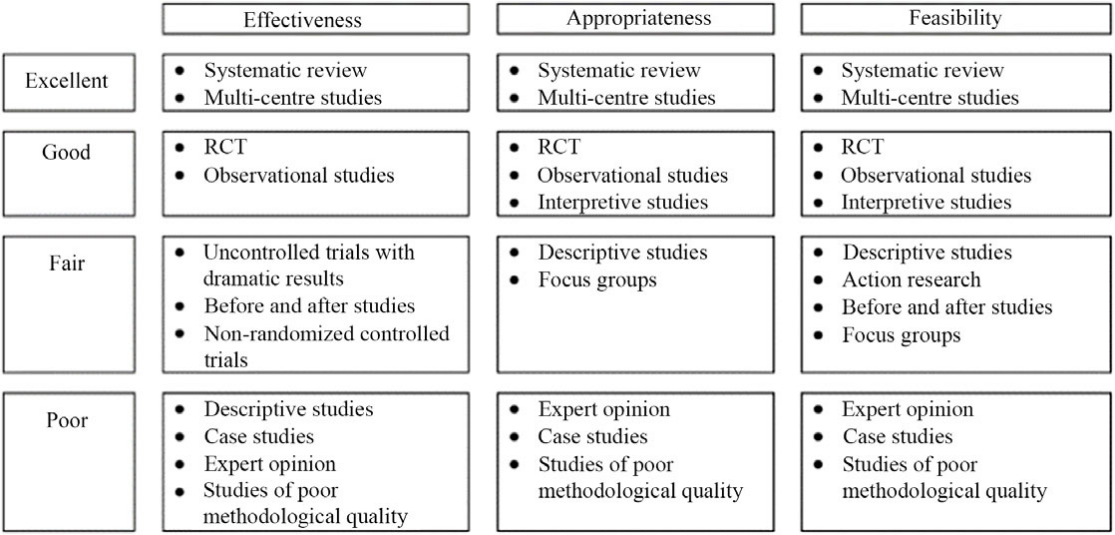
An example of a framework for ranking evidence evaluating healthcare interventions. Taken with permission from^(13)^.

Research designs offer different levels of confidence about the results of the research, and the idea of a hierarchy of evidence has been used widely. Hierarchies of evidence describing the efficacy of health interventions are often drawn as pyramids, showing weaker study designs at the bottom and stronger study designs at the top. At least 80 different hierarchies of evidence have been identified^(10)^, and most follow a similar order, focusing on the ability of the study design to test the efficacy of interventions in humans. ‘Expert opinion’ and ‘mechanistic’ research performed *in vitro* or in animal models (as opposed to mechanistic studies in human ‘randomised controlled trials’ (RCT)) are placed at the bottom, followed by ‘observations in individuals or small groups of humans’ (e.g. case reports, case series), followed by ‘observational studies’ in the middle (case–control (retrospective), cohort (prospective)), followed by ‘RCT’ and finally ‘systematic reviews and meta-analysis of RCT at the very top (Fig. 2(a)). For an intervention to be useful it must be both efficacious (i.e. able to produce the desired result under controlled, ideal circumstances) and effective (i.e. able to produce the desired result in the ‘real-world’ conditions while considering acceptability, adherence and feasibility)^(11)^. The study designs required to assess efficacy and effectiveness are necessarily distinct, and therefore, a combination of study designs is typically required to thoroughly understand the implementation and impact of an intervention. Therefore, the ‘best’ method by which to acquire evidence depends on the research question^(12)^. Nevertheless, hierarchies of the most appropriate study designs remain part of the quality framework with adequately powered multi-centre trials and systematic reviews of any kind of study design considered to provide the most powerful evidence (Fig. 3)^(13)^. Existing hierarchical schemes also do not refer to emerging or novel research methods, such as pragmatic trials, implementation science and real-world health data.

It is crucial that consideration is given to the purpose of the research, as well as the strengths and limitations, interpretation and misinterpretation of any hierarchy of evidence used. It is important to note that study designs ranked lowest in any hierarchy may still be the best available for the required purpose of the research. Guidelines necessarily must use the ‘best available evidence’, and this may include low-quality studies if these are the only studies available. It is also important to acknowledge that methods that are inappropriate for assessing efficacy, may still be highly appropriate for assessing some aspects of effectiveness such as feasibility or acceptability (Fig. 3). For example, randomised trials have demonstrated that exclusive enteral nutrition is an effective treatment for active Crohn’s disease, especially in children, but may also be used in adults who wish to reduce steroid exposure^(15)^. However, in adults a cross-sectional survey of clinical case notes has shown that in practice exclusive enteral nutrition is commonly ceased early due to poor compliance^(16)^ and qualitative interviews with patients with Crohn’s disease have reported poorer acceptability due to social restrictions and dietary monotony^(17)^. Therefore, although cross-sectional surveys and qualitative interviews would provide poor evidence of the effectiveness of an intervention, they may considerably improve understanding of feasibility and acceptability and contribute to improving the effectiveness of interventions when applied in practice (Fig. 3).

Mechanistic studies are particularly important in determining whether findings from observational cohort studies can be strengthened by supportive evidence for plausible biochemical or physiological mechanisms. They also increase the certainty of evidence from RCT by demonstrating observed effects are operating through a well-understood pathway^(18)^.

Observational studies, including large and diverse populations, provide evidence of effects at scale and over long periods^(19)^ and may be the only practicable form of assessing the potential impact of a particular diet or nutrient (the exposure) on a health outcome, where an RCT is not feasible. For example, an RCT intervention may require unfeasibly lengthy follow-up (e.g. effect of fibre on risk of mortality from colorectal cancer), result in unacceptable ethical issues (e.g. where a ‘no intervention’ would be ethically unjust) or is too costly to undertake. These challenges mean that as well as use of cohort data, it is sometimes necessary, though not ideal, to use ‘real-world’ evidence to inform practice. Maduri *et al.* (2021) discuss this in relation to the use of ‘artificial intelligence’ to make predictions from real-world data collected from patient groups.

These mechanistic and observational studies can make some contribution to supporting the findings from intervention studies when RCT are few in number or low in quality. Not all RCT are well-designed and therefore a poorly designed RCT (e.g. one that is underpowered, poorly blinded, incompletely analysed, with high risk of bias) should not be automatically considered superior to a well-designed cohort study (e.g. one that is adequately powered and includes comprehensive assessment of incident disease, high-quality measurement of nutrient intake, extensive adjustment for confounding variables). Critical appraisal of the individual study methods is required to make these judgements, and this is discussed in the next section. Clinical guidelines increasingly differentiate between higher- and lower-quality study methods in making recommendations (discussed later in this paper). Some hierarchies of evidence have been modified to acknowledge the variation in quality (Fig. 2(b)).

The place of systematic reviews at the top of hierarchies of evidence has also been challenged, because the amalgamated findings are only as good as the rigour of the systematic review itself (strength of search terms, comprehensive search strategy, relevant eligibility criteria etc) and the design of the individual studies included. However, a systematic review of case-control studies (a weak study design) cannot provide the same level of evidence certainty as a systematic review of RCT^(14)^. Therefore, modified hierarchies of evidence that place systematic reviews as a method of analysing other methodologies have been proposed (Fig. 2(c))^(14)^.

N-of-1 trials involve an individual undergoing routine care who is allocated to both intervention and control, and in the most rigorous design, these are delivered in random order and blinded to both the participant and the researcher, while response is carefully monitored. N-of-1 trials have been proposed in nutrition and dietetic practice^(6,20)^ as they can account for personalised responses to intervention that an RCT in a large population cannot and of course can be performed at limited cost in the absence of an available RCT^(21)^. However, such trials are rarely performed in routine clinical practice due to challenges of blinding interventions to both participant and researcher and the time to complete the trials means they are largely restricted to chronic disorders^(6)^.

Finally, qualitative research (e.g. semi-structured interviews and focus groups) and mixed methods are increasingly used in nutrition and dietetics. Although the purpose of qualitative research is not to measure the quantitative impact of an intervention, but rather to explore experience and perceptions, it is crucial in understanding acceptability and feasibility in routine care, an essential component of evidence-based practice^(22)^ and included explicitly in some hierarchies (Fig. 3).

#### Assessing the quality of study methods

As well as understanding the optimal methodological study design, critical appraisal should also involve understanding the strengths and limitations of the individual study methods and their risk of bias. RCT have the lowest risk of bias but there are some unique challenges that are hard to overcome in nutrition and dietetic research, including conducting the ‘gold-standard’ RCT.

Firstly, true blinding can be difficult to achieve in studies of diet or foods because of the difficulties in providing adequate controls. Placebos are possible for micronutrient supplementation trials, but much more difficult to develop for food supplementation or diet modification studies^(23)^. Domiciled feeding studies or complete meal provision studies may be able to overcome blinding issues but are extremely expensive and do not reflect how dietary change would occur in real life^(24)^. Sham dietary advice is an alternative (and can be ethical in certain circumstances) but must ensure that the sham advice is similar in complexity and cost to the intervention diet, and yet does not result in significant change to nutrient intake^(25)^. Alternatively, ‘standard care’ can be used in the form of basic nutrition information (e.g. general healthy eating advice), but if such advice could affect the outcome then the study would need to be comparative and ideally using an RCT^(26)^.

Secondly, adherence to either intervention or comparison is rarely 100 % even with a simple intervention such as supplements, and therefore, the threshold for an effect may not be reached and thus the expected effect size may not be achieved. Extensive approaches to improving adherence should be included in dietary research including offering alternative dietary options to satisfy personal, cultural and religious preferences and to involve potential participants in the design of the research. These studies require significant resource and skilled individuals which greatly increase their costs.

Third, diet modification studies that change one component (e.g. low carbohydrate diet) will inevitably affect others (e.g. increase fat, reduce fibre) and therefore the nutrient responsible for the effect often cannot be identified. Dietary collinearity (the positive or negative association of the intake of one nutrient with another) is an important potential confounder in research studies and should be carefully monitored^(23)^. Collinearity may be particularly important in whole diet interventions that intervene with several potentially synergistic components, rather than isolated nutrient or food interventions^(23)^. For example, a study of a gluten-free diet may result in some participants doing more cooking from scratch (improving the nutritional profile of foods eaten), whereas in other participants it may encourage them to purchase pre-prepared meals labelled as gluten-free (increasing the intake of relatively energy-dense and micronutrient poor foods). This introduces variability into the intervention which may reduce the power and efficacy of the study.

Many tools have been developed to assist in the critical appraisal of different study designs and study methods as shown in Table 2 ^(29)^, but despite the specific challenges in nutrition and dietetics there are few tools designed for nutrition trials. However, review papers that raise these issues can be a useful resource to support the evidence-based practitioner in evaluating challenging aspects of critically appraising the methods of dietary intervention studies^(23–25,30)^.

**Table 2.** Examples of critical appraisal tools for use with different study designs

Study design and critical appraisal tool	Website
Systematic review & meta-analysis	
AMSTAR-2	https://amstar.ca/Amstar_Checklist.php ^(27)^
CASP Checklist for Systematic Reviews	https://casp-UKnet/casp-tools-checklists/
NICE Methodology Checklist for Systematic Review	https://www.nice.org.UK/process/pmg6/resources/the-guidelines-manual-appendices-bi-2549703709/chapter/appendix-b-methodology-checklist-systematic-reviews-and-meta-analyses
Randomised controlled trials (RCT)	
Cochrane risk-of-bias tool for randomised trials (RoB-2)	https://training.cochrane.org/handbook/current/chapter-08 ^(28)^
CASP Checklist for RCT	https://casp-UKnet/casp-tools-checklists/
NICE Methodology Checklist for RCT	https://www.nice.org.UK/process/pmg6/resources/the-guidelines-manual-appendices-bi-2549703709/chapter/appendix-c-methodology-checklist-randomised-controlled-trials
Cohort studies	
CASP Checklist for cohort studies	https://casp-UKnet/casp-tools-checklists/
NICE Methodology Checklist for Cohort Study	https://www.nice.org.UK/process/pmg6/resources/the-guidelines-manual-appendices-bi-2549703709/chapter/appendix-d-methodology-checklist-cohort-studies
Case-control studies	
CASP Checklist for case-control studies	https://casp-UKnet/casp-tools-checklists/
NICE Methodology Checklist for case-control studies	https://www.nice.org.UK/process/pmg6/resources/the-guidelines-manual-appendices-bi-2549703709/chapter/appendix-e-methodology-checklist-casecontrol-studies
Qualitative research	
CASP Checklist for qualitative studies	https://casp-UKnet/casp-tools-checklists/
NICE Methodology Checklist for qualitative studies	https://www.nice.org.UK/process/pmg6/resources/the-guidelines-manual-appendices-bi-2549703709/chapter/appendix-h-methodology-checklist-qualitative-studies

AMSTAR-2, A MeaSurement Tool to Assess systematic Reviews; CASP, Critical Appraisal Skills Programme; NICE, National Institute for Health and Care Excellence; ROB-2, Risk of Bias-2.

Experts in nutrition and dietetics, such as dietitians and nutritionists, should have skills in research design, conduct and analysis, and evidence-based practice including critical appraisal. Competency in these areas is included in standards set by professional associations or governing bodies across the globe, including Australia^(31)^, Canada^(32)^, the UK^(33,34)^ and USA^(35)^. Despite this, studies report variable levels of research involvement^(36)^ and application of evidence-based practice among dietitians^(37)^, with few studies specifically in nutritionists. Studies consistently cite barriers including a lack of time and funding, and lack of confidence in research and evidence-based practice. Approaches are needed to increase education and involvement in research and evidence-based practice during university programmes in nutrition or dietetics^(38)^ and once in practice^(39)^, as well as improving research collaboration and funding in nutrition^(40)^.

#### Conflicts of interest and sponsorship

Bias within research studies can arise for several reasons as discussed already, but conflicts of interest, especially from sponsorship by industry partners, has attracted particular attention. The food and health sectors are significant global economic actors, that encompass agriculture, food processing, distribution and retail, with an estimated revenue of $8·77 trillion in 2022 and rising (Statista.com). Thus, the careful management of potential conflicts of interest is needed to assure high-quality science by the researcher and independence of the findings by the sponsor. For example, most university contracts with industry include a clause to allow publication of the findings, whatever the outcome of the research, and rigorous legislation exists to ensure health claims on food are supported by high-quality evidence^(2)^.

Nevertheless, vested interests linked to industry sponsorship can contribute to bias in research. Systematic review evidence suggests that bias can exist in interventions involving potential new treatments, resulting in outcomes that could favour the sponsor, but is not necessarily greater in food industry-sponsored studies. Evidence specific to artificial sweetened beverage effects on weight is one case where industry sponsorship has been reported to cause bias to favour conclusions in support of the industry position^(41)^. Where the sponsor, investigator and participant are blinded to the intervention, bias can be minimised, and aforementioned contractual obligations and trial registration can prevent sponsors from selectively reporting favourable results^(42,43)^.

Despite the potential for bias, the food industry can offer considerable expertise and innovation to seek solutions for challenges in the food and health system. In the context of research trials, many researchers choose to involve an industry sponsor because this allows the production of a high-quality palatable product that is more likely to be acceptable to patients or volunteers. Acceptability of a food item or diet is an absolute requirement for a long-term intervention study.

#### Assessing certainty using the GRADE method

There are various methods to assess certainty of a collective body of evidence. The GRADE method (Grading of Recommendations, Assessment, Development and Evaluation) is a widely used framework that helps researchers and guideline developers *rate* evidence quality in studies and *grade* the strength of guideline recommendations^(44,45)^. The intent is to determine the level of certainty in the evidence, whether the intervention or exposure is effective or not. How evidence is rated has potentially substantial implications as to how knowledge is applied in clinical practice, hence a rigorous and widely accepted assessment tool like GRADE is critical to produce high-quality systematic reviews and guidelines. The GRADE method assigns four levels for evidence certainty: high, moderate, low and very low^(46)^. When using GRADE in systematic reviews, ‘Summary of Findings’ tables are developed and certainty of evidence is determined^(47)^.

The GRADE’s evidence-to-decision framework is used to consider evidence and other specific elements in formulating guideline recommendations^(48)^. GRADE classifies guideline recommendations as strong or weak. A strong recommendation indicates that most patients should receive the intervention as the recommended course of action, while a weak recommendation may mean that different choices will be appropriate for different patients based on patients’ values and preferences. Different recommendation examples are shared later in the section on the Evidence Analysis Library (EAL).

Although judgments and deliberation are required, the systematic and transparent GRADE approach provides a structured framework to engage in the necessary judgements and reach rating decisions. To apply the GRADE method, specialised training is recommended. As a starting point, Cochrane offers online introductory courses and resources to learn more about the GRADE method and create summaries of findings (https://training.cochrane.org/). The most comprehensive resource on the GRADE method is the GRADE handbook^(48)^.

### Implementation of nutrition research in practice: available national and international guidelines

Clinical practice guidelines are a vehicle for integrating research evidence with clinical expertise and patient values, in order to guide practitioner and patient decisions and optimise patient care^(49)^. The quality of clinical practice guidelines and the practice guidance contained therein is variable owing to both the quality of the evidence available to support the recommendations and the quality of the guideline development process. The recognised process for creating clinical practice guidelines has evolved over the last few decades from expert deliberations informed by narrative literature reviews, to more rigorous, transparent and evidence-based approaches^(50,51)^. Even so, there is no universally accepted standard approach to guideline development and groups frequently create internal processes specific to their own needs and local context^(49)^. To help users critically appraise guideline quality and assess their usefulness, an international team of guideline developers and researchers known as the AGREE Collaboration (Appraisal of Guidelines, Research and Evaluation) created a validated instrument known as the AGREE II to assess six domains of guideline quality: scope and practice, stakeholder involvement, rigour of development, clarity of presentation, applicability and editorial independence^(52,53)^. Recently, the AGREE II instrument has been used to compare the quality of multiple clinical practice guidelines related to a single topic, such as clinical nutrition for critically ill adult patients^(54)^ or nutrition management for patients with Covid-19^(55)^.

Globally, there are many scientific groups working to develop nutrition-focussed practice guidelines to help inform individualised nutrition care. Nutrition-focussed guidelines have been produced at the intergovernmental level by the WHO^(56)^, the governmental level by the National Institute of Health and Care Excellence in the UK^(57)^, by specialised societies and multi-professional organisations such as European Society of Clinical Nutrition and Metabolism^(58)^ and European Society for Paediatric Gastroenterology, Hepatology and Nutrition^(59)^, by health charities such as Diabetes UK^(60)^ and as part of the work of national dietetic associations, such as the British Dietetic Association^(61)^ and the EAL from the Academy of Nutrition and Dietetics (AND) in the USA^(62)^. How these organisations develop nutrition-focussed practice guidelines has been compared in Table 3, using the AGREE II criteria. Finally, there are also groups dedicated to working with existing nutrition-focussed practice guidelines, helping to further translate the information they contain into practical recommendations that dietitians and other nutrition professionals can use when working with individual clients. One such collaboration is Practice-based Evidence in Nutrition(R) (PEN), which uses the principles of evidence-based practice to create contextualised practice recommendations, toolkits and client-facing handouts.

**Table 3.** Comparison of clinical practice guideline development processes using the Appraisal of Guidelines for REsearch & Evaluation (AGREE) II criteria

AGREE II criterion	WHO^*^	National Institute of Health and Care Excellence (NICE)^†^	European Society of Clinical Nutrition and Metabolism (ESPEN)^‡^	Academy of Nutrition and Dietetics (AND)
Scope & Practice	Guideline groups are asked to identify clear, actionable objectives for the guidelines and research questions that are framed in a way that enables a systematic literature search (e.g. PICO)	Guideline groups are not provided specific direction regarding setting guideline objectives but determine the target population as part of the scoping process. Specific questions are developed using an available framework (e.g. PIC, SPICE, PICOT etc).	Guideline groups are not provided specific direction regarding setting guideline objectives. Specific questions are developed using the PICO format, and in this way, the target population for the guideline is defined.	The scope of the guideline is determined and used as the framework to execute the supporting systematic review. All guidelines are supported by systematic reviews. Systematic review questions are framed considering the NCP model and conform with PICOT format^(63)^.
Stakeholder Involvement	The end user as well as the service users and individuals whose health and well-being will be affected by the guidelines must be identified early so that representatives from these groups can be part of the Guideline Development Group. Additionally, the GDG includes technical experts and must represent diverse perspectives with adequate geographic and gender representation.	Guideline groups must be multidisciplinary with representation from practitioners, professionals, providers, commissioners, researchers and lay members with relevant experience..	The guideline working group is determined by the Guidelines Editorial Board and is based primarily on academic expertise but with consideration for gender balance, geographic location and prior experience in developing guidelines.	Subject matter experts vote on recommendation statements. A blinded interdisciplinary external review is conducted with 8–10 subject experts. Names and credentials are listed. Further stakeholder input is sought using targeted or open public comment or focus groups. Individuals or groups may provide specific comments to the drafted recommendations.
Rigour of Development	Recommendations are developed based on a systematic review of the literature using explicit methods. The GRADE approach is used to assess the overall evidence quality and to develop recommendations. Guidelines must undergo external peer review before publication and should include a ‘review by’ date but need not include a procedure for update.	Recommendations are developed based on a systematic, transparent and reproducible process for searching, screening and reviewing the evidence. The GRADE approach is recommended, but other critical appraisal frameworks may be approved in advance. Draft guidelines are posted for consultation with registered stakeholders and there is a clear process for updates.	Recommendations are developed based on a systematic review of the literature with documentation. Publications are classified according to evidence level, recommendation grade and form of recommendation during the Scottish Intercollegiate Guideline Network grading system. Guideline recommendations are published open access in ESPEN’s journal, but are not externally reviewed as part of that process.	Systematic review questions are investigated according to the AND’s methodology^(64)^ which includes the GRADE methodology and PRISMA guidelines^(65,66)^. Evidence is considered in the context of NCP and GRADE’s evidence-to-decision framework., as well as feasibility and resource use. Recommendations are designed to be clear, specific, structured, transparent and actionable.
Clarity of Presentation	Each recommendation is accompanied by: 1. Strength of the recommendation (strong or conditional/weak), including justification 2. Quality of the evidence on which they are based 3. A set of remarks that explain context for understanding and implementation 4. Summary of the evidence	Recommendations are action-oriented, precise, concise, person-centred and use wording that reflects the strength of the recommendation (e.g. must *v*. offer *v*. consider).	Each recommendation is accompanied by: 1. Evidence Level 2. Recommendation Grade 3. Form of Recommendation, and 4. Classification of the strength of consensus	Recommendations are ‘operationalised’ using the nutrition care process, related terminology and informatics tools. Each guideline includes five major components: 1) executive summary 2) introduction 3) scope 4) major recommendations 5) dissemination and implementation.
Applicability	Factors affecting feasibility, implementation and resource implications are considered as part of the GRADE process. Guidelines should include outcome measures that can be used to monitor effectiveness and impact.	Levers and barriers to guideline implementation are considered and the guideline group works with NICE teams to produce tools and resources that support implementation. A requirement for criteria used to monitor the guidelines is not described in the manual.	The Standard Operating Procedures for guidelines do not mention an assessment of facilitators or barriers to using the guidelines, an exploration of resource implications or monitoring criteria. There is a section that suggests activities and tools that support guideline implementation.	Stakeholder input highlights concerns surrounding feasibility of the recommendations, patient-centred language and other important factors. Ongoing evaluation of the guidelines’ use supports adoption and allows monitoring of application
Editorial Independence	Guideline funders cannot participate in the development process or influence the recommendations. All members of the guideline group must complete a declaration of interests form and there is a process for managing conflicts of interest.	All members of the guideline group are required to declare potential conflicts of interest, which are managed in accordance with a NICE policy.	Guidelines are funded by ESPEN and may be supported indirectly by industry funding through unrestricted grants. All group members declare a competing interests before participation but there is no description of how conflicts are managed. Pharmaceutical employees are excluded from the systematic literature review and consensus meetings.	Guidelines do not receive industry funding and the AND is the primary source of support. Inter-agency partnerships are sought, where possible, to reduce duplication and maximise efficiency of resources. Examples include National Kidney Foundation, the American Council on Exercise and Dietetic Practice Groups of AND.

PICO = population, intervention, comparator, outcome; PIC=population, intervention, comparator; SPICE = setting, perspective, intervention, comparison, evaluation; PICOT=population, intervention, comparator, outcome, treatment; NCP = Nutrition Care Process; GRADE = Grading of Recommendations, Assessment, Development and Evaluations; PRISMA = Preferred Reporting Items for Systematic Reviews and Meta-Analyses.

* World Health Organization. WHO Handbook for Guideline Development – 2nd ed. 2014 Dec 14. Available from: https://www.who.int/publications/i/item/9789241548960.

† National Institute for Health Care Excellence. Developing NICE guidelines: the manual (PMG20). Process and methods. 2014 Oct 31 (last updated 2022 Jan 18). Accessed 2022 Aug 22. Available from: https://www.nice.org.UK/process/pmg20/chapter/introduction.

‡ Bischoff SC, Singer P, Koller M, Barazzoni R, Cederholm T, *et al.* Standard operating procedures for ESPEN guidelines and consensus papers. Clin Nutr. 2015;34:1043–41. Available from: https://www.espen.org/files/ESPEN-Guidelines/0__Standard_operating_procedures_for_ESPEN_guidelines_and_consensus_papers_2.pdf.

As examples of two methods of applying the evidence to practice, the EAL and PEN System are described in more detail below. The aim of a well-defined methodological process is to promote objectivity, transparency and reproducibility while minimising issues like conflict of interest.

#### Evidence Analysis Library® of the academy of nutrition and dietetics

**Fig. 4.**
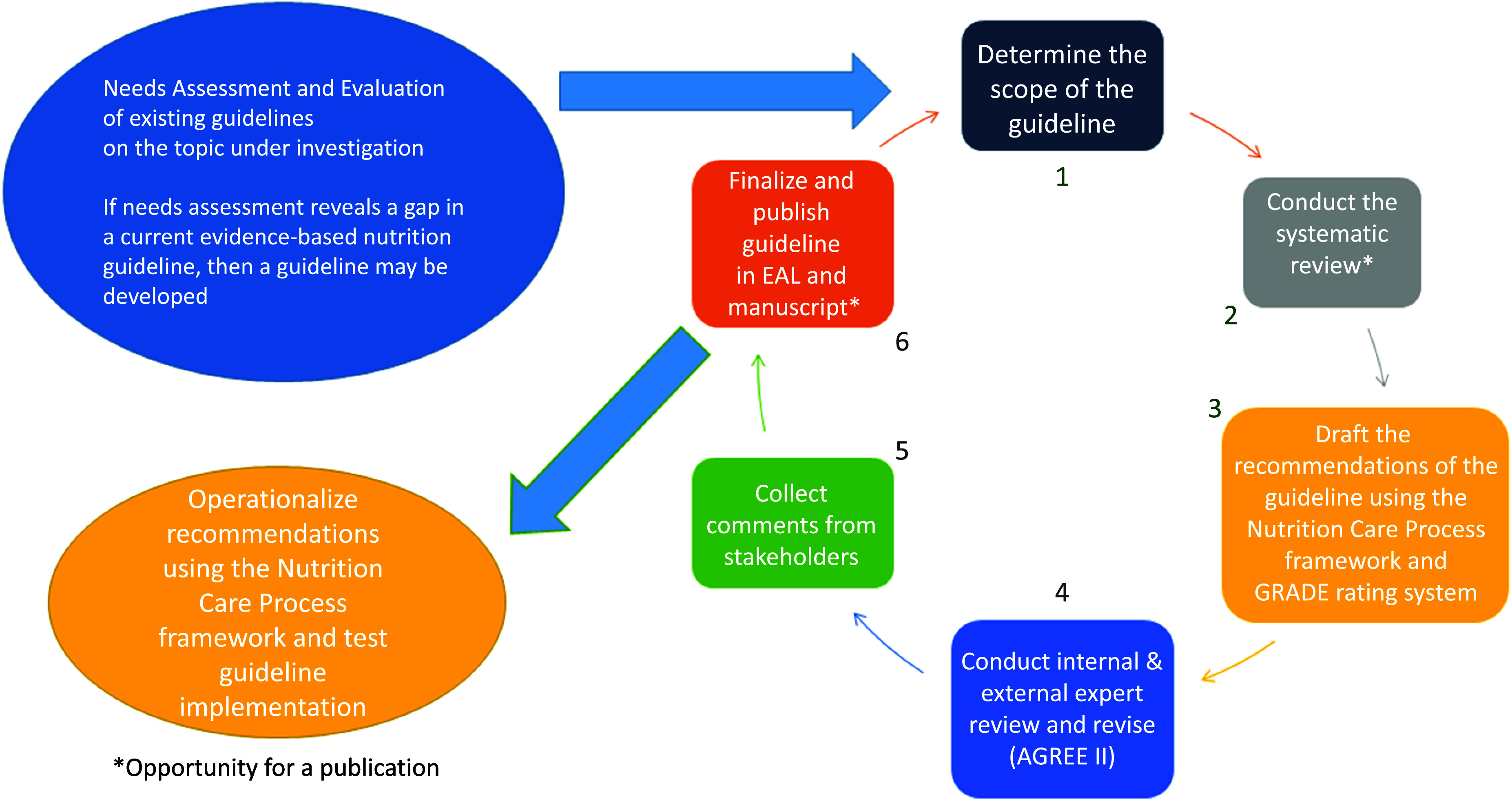
Evidence analysis library guideline development process. This rigorous and transparent multi-step method to develop guidelines^(62)^ is described in detail in a series of learning modules (in five short videos) (www.andeal.org/tutorials).

In this section, we describe the guideline development methods of the AND^(62,65)^. Since 2004, the AND has published evidence-based nutrition practice guidelines (referred to as guidelines thereafter) to support the practice of nutrition and dietetic professionals who strive to provide evidence-based, effective and safe nutrition care. The source of funding is primarily AND whose guidelines do not receive industry funding. The guidelines are a collection of action-oriented practice recommendation statements that are based on a systematic review^(64)^, using a process which integrates the GRADE method. The guidelines are organised by the nutrition care process model and may focus on one or more steps of this process^(63)^. The nutrition care process model encourages people-centred care and ongoing research of nutrition care outcomes. The guideline development method is summarised in Fig. 4 and further details are provided in Table 4. A comprehensive evidence analysis manual describes detailed workflows and provides forms for evidence synthesis projects^(67)^.

**Table 4.** Five components of evidence-based nutrition guidelines produced by the Evidence Analysis Library, academy of nutrition and dietetics

Executive summary	Includes a list of all the recommendations and evidence ratings within a guideline
Introduction	Describes the topic overview, development and any approved external guidelines
Scope	Provides the purpose, guideline category, clinical specialties, intended users, objectives, target population, interventions and practices considered. The scope encompasses important technical subsections, and these are: a statement of intent, general and specific methods with links to systematic review search plans and respective results. Search plans provide dates of literature searches, inclusion and exclusion criteria, search terms, databases searched and lists of included and excluded articles
Major recommendations	Includes a list of recommendation topics organised by the Nutrition Care Process. Recommendations describe the rationale, risks and harms of implementing the recommendation, conditions of application and recommendation strength rationale
Dissemination and implementation	Provides an updated list of resources and events from the project including published manuscripts, webinar series, national and local events, education resources for use in the education and training of dietetic interns and students, speakers guide (ready-to-use slide decks ideal for in-service trainings and professional meeting presentations), information about implementation activities such as studies, and patient resources when applicable

The selection of topics for new guidelines is determined by criteria established by the AND’s Council on Research and includes a needs assessment and evaluation component of existing guidelines. Typically, a guideline team comprises methodologist, lead analyst, project manager, six to eight subject experts and patient advocate and/or patient organisation representative whenever possible.

Recommendations are rated using the GRADE rating system (1), strong (we recommend); (2) weak (we suggest)) and the certainty of evidence assigned according to the evidence the recommendation is based on (A, high; B, moderate; C, low; D, very low), for example 1A means a strong recommendation based on a high certainty of the evidence^(65,68,69)^. For example:

*In adults with chronic kidney disease stages 3–5 who are metabolically stable, we recommend, under close clinical supervision, protein restriction with or without keto acid analogs, to reduce risk for end stage kidney disease or death (1A).*

An example of a weak recommendation based on low certainty of evidence would be:

*‘In adults with chronic kidney disease stages 3–5, including those on chronic dialysis, we suggest the use of a 3-day food record, conducted during both dialysis and non-dialysis treatment days (when applicable), as a preferred method to assess dietary intake (2C)’.*

The process consists of an internal and external review of the draft guideline (Table 4 & Fig. 4). Reviewers include, but are not limited to, nutrition and dietetics practitioners, physicians, nurses, pharmacists and psychologists. The AGREE II instrument^(70)^ is used for evaluation. Any recommendation statements changed during the review process are voted on and approved by the subject matter experts on the project.

Following external review, other stakeholders may highlight concerns surrounding feasibility of the recommendations, patient-centred language and other important factors. In recent guideline projects, to obtain end user feedback on the applicability of a guideline, practitioners were recruited, provided with a general guideline orientation and asked to test the guideline in their practice for two weeks. Recruited practitioners then came together in a focus group to describe their own experiences and their clients’ feedback. The project team reviews the feedback and addresses comments while maintaining the integrity of the evidence. The goal is to generate a comprehensive guideline (as summarised in Table 4) and a practitioner guide (shortened version or infographic, that uses language that can help communicate research to clients and is intended for use with clients for shared decision-making). An example practitioner guide (on saturated fat) can be found here: https://www.andeal.org/files/files/Saturated%20Fat/2023–07_DLM-SF_PractitionerGuide_2023_01_25.pdf and the respective comprehensive guideline is here: https://www.andeal.org/topic.cfm?menu=3693&cat=6214. There is a growing emphasis on developing information on ‘implementation considerations’ that are rich and detailed with tools and pragmatic suggestions. Guideline implementation may need aligned efforts between stakeholders and systems-focused approaches to implement change at the organisational level. To assist practitioners with these considerations, the EAL provides an implementation guide manual (that can be found here: https://www.andeal.org/vault/2440/web/files/EAL/EAL_Guideline_ImplementationManual_2022Nov.pdf)

The AND Council on Research provides final approval that the EAL project team has appropriately addressed all the comments received. The final guideline is then published on the EAL subscription website (www.andeal.org). Guidelines and supporting systematic reviews are also published as separate manuscripts in peer-reviewed journals. The AND’s guidelines are recognised and referenced by the Guidelines International Network (https://g-i-n.net/).

Historically, the AND guidelines are revisited every five years. A scoping review is carried out to help inform authoritatively whether substantial literature has been published on the topic since the last systematic review. The Council on Research, supported by the EAL staff, determines which recommendations of the guideline should be revised. To reduce duplication and maximise efficiency of resources inter-agency partnerships are sought. As an example, the recent update of the chronic kidney disease guideline was completed in collaboration with the National Kidney Foundation. It describes 83 recommendations on important nutrition topics in chronic kidney disease, including nutrition screening and assessment; medical nutrition therapy; protein and energy intake; micronutrients; electrolytes; nutritional supplementation; and dietary patterns (Handu *et al.* 2021).

Recently, EAL staff have followed the stages of GRADE-ADOLOPMENT. Adolopment here refers to evaluating and then adopting ‘as is’ or adapting existing guidelines instead of creating guidelines from the ground up. The GRADE-ADOLOPMENT process helps researchers decide transparently and systematically whether it is appropriate to adapt or adopt existing recommendations from other organisations in order to expedite the generation of recommendations that are current, rigorous and evidence-based^(71)^.

Wide dissemination and implementation of guidelines is necessary to deliver quality care and improve targeted health outcomes. To this end, the AND conducts research on the use of guidelines and resulting outcomes. This is an emerging process in which the recommendations are ‘operationalised’ using the nutrition care process, related terminology and informatics tools^(63,72–74)^. Such efforts intend to support guideline adoption and allow longitudinal monitoring of guideline application.

#### Practice-based Evidence in Nutrition® (PEN)

The PEN System is an online nutrition knowledge translation platform that is jointly managed by Dietitians of Canada, the British Dietetic Association and Dietitians Australia^(75)^. It is funded by subscription revenue and does not accept funding from industry or advertising. Developed in 2005 as a way of creating and managing a dynamic, online clinical nutrition manual, it has now grown to provide evidence-based practice guidance for more than 1000 practice questions in over 200 topic areas, including those related to population health, health conditions, food and nutrients and professional practice. The PEN team does not create independent clinical practice guidelines, but rather develops practice recommendations using the best evidence available. This evidence might arise from clinical practice guidelines (or sometimes multiple sets of guidelines developed by different groups), or it might come from secondary research studies, primary research studies, grey literature that provides additional practice context or a combination thereof. Using this adaptable approach to seek the ‘best available evidence’ the PEN System aims to enhance evidence use by practitioners. Content on the PEN System is internationally peer-reviewed by academic and practice-based experts.

**Fig. 5.**
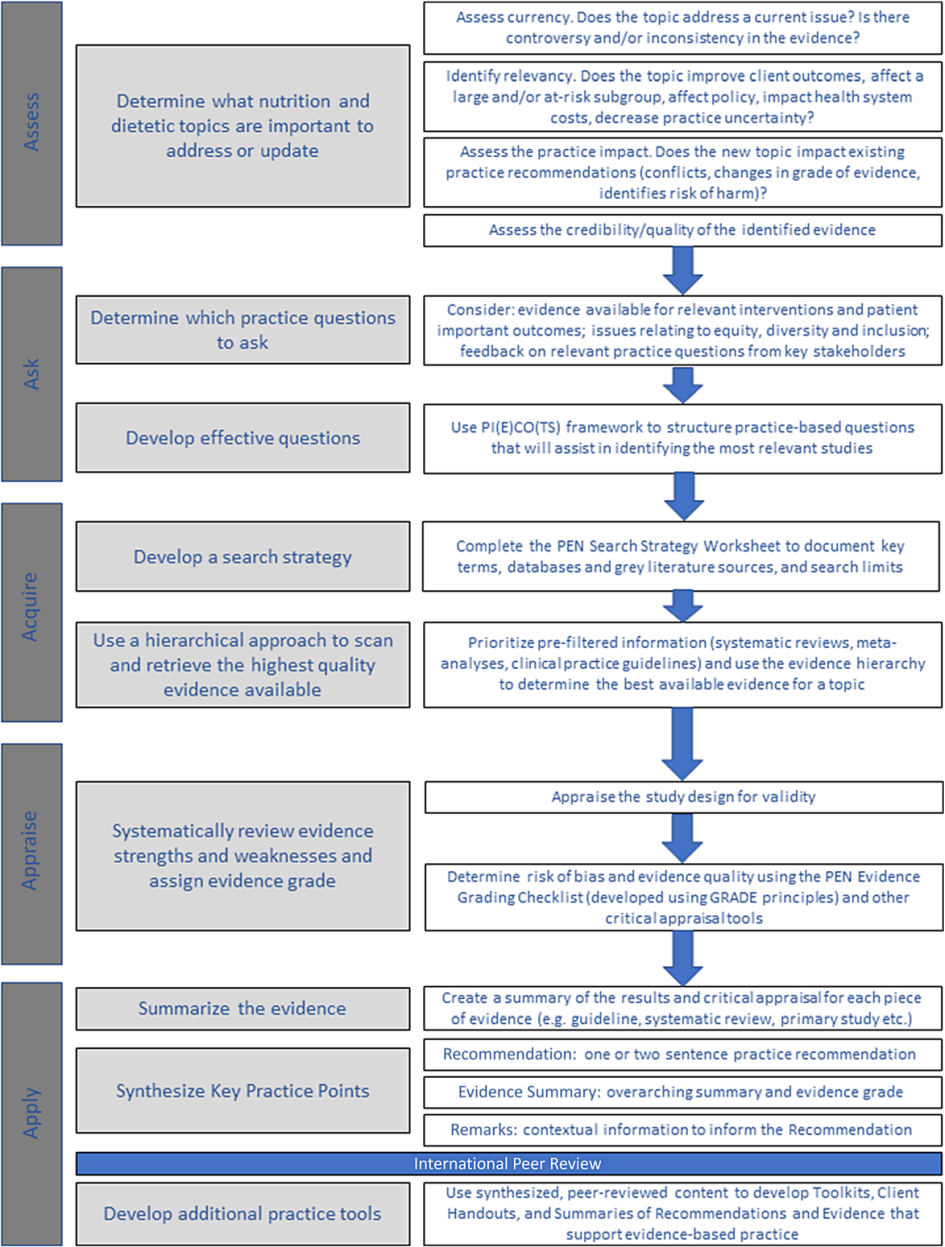
Schematic representation of the PEN process for developing practice recommendations and tools. PI(E)CO(TS): Population or Problem, Intervention or Exposure, Comparison, Outcome, Timeframe, Setting or Study Design.

Practice guidance on the PEN System is developed using the five ‘A’s model of evidence-based practice^(5,76)^: assess, ask, acquire, appraise and apply (Fig. 1(b)). An overview of the PEN process is provided in Fig. 5. The first ‘assess’ stage uses an algorithm developed for this purpose^(77)^. In the second stage (Ask), ‘foreground’ questions are emphasised, that is questions that address issues of care and/or decision-making and focus on assessment, treatment and prevention, rather than ‘background’ questions, which focus on aetiology, prevalence, incidence, prognosis or disease course^(78)^. The ‘acquire’ stage uses a systematic approach executed by an Evidence Analyst^(79)^. The appraisal stage uses an Evidence Grading Checklist^(80)^, which categorises evidence on a scale of A to D. A-level conclusions are supported by GOOD evidence, B-level conclusions supported by FAIR evidence, C-level conclusions supported by LIMITED evidence or expert opinion and for a D-level rating conclusions cannot be drawn or extremely limited because evidence is unavailable, poor quality or contradictory.

An example of a practice question with Key Practice Points is shown in box 2, including a recommendation, summary of the evidence and remarks that identify implementation considerations or help contextualise the recommendation for different regions, settings or sub-populations^(81)^. In addition, summaries and critical appraisal of the individual articles used to create the recommendation, and comments and rationale are provided. These provide further information to support practice (e.g. food sources of a nutrient of interest) or proposed mechanisms of action.

Box 2. An example of a practice question with Key Practice Points.

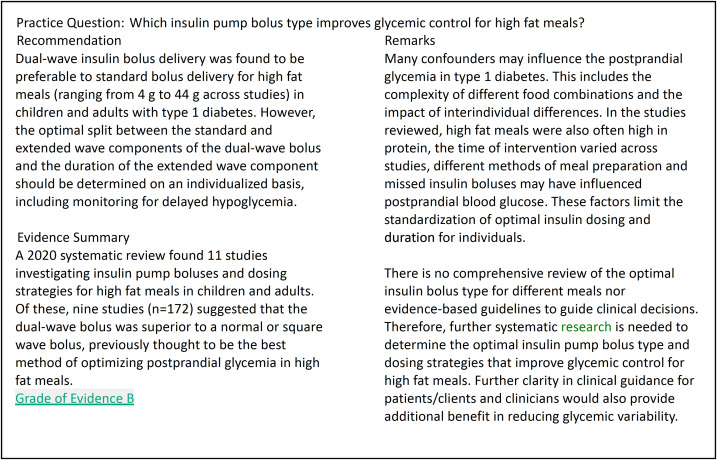

Finalised content is further translated into knowledge products designed to support practice, such as client-focussed handouts and practice guidance toolkits, which outline key considerations related to the assessment, diagnosis, intervention and monitoring of health conditions in a format consistent with nutrition care process terminology^(82)^.

EAL and PEN offer two examples of guideline development for nutrition interventions and illustrate the significant progress made in the last decade, but there is less certainty about how or whether practitioners adopt and use guidelines. There is some evidence that guidelines are not implemented fully in practice and further research to understand the barriers and facilitators to guideline implementation is needed. As mentioned in the previous section, this is now one focus of the AND work programme.

### The influence of other sources of nutrition information and misinformation

Evidence-based clinical practice guidelines are not the only source of information used to guide decision-making in what people eat and drink to improve their health or manage their condition. Other information comes from a variety of sources, such as patients and their family members, advertisements, non-qualified practitioners, social media (including influencers and celebrities), internet sites, media outputs and urban myths. These sources can lack accuracy and reliability, opinion is not always based on science^(83)^, and this area is largely unregulated, in contrast to food claims made by industry^(2)^. Such misinformation can be harmful because it can lead people to make poor dietary choices that may negatively affect their health.

Some of the factors that contribute to the spread of nutrition misinformation include the abundance of conflicting information online, the proliferation of fad diets and health products, and the tendency for sensational headlines to attract more clicks, views and sales. Several studies have shown varying levels of quality in the content of nutrition-related articles in newspapers^(84,85)^, which remain an important source of information for the public. As nutrition misinformation continues to spread through various channels, it becomes increasingly important for healthcare practitioners to be aware of its impact on patients and to take steps to mitigate its effects.

These factors add complexity to implementing evidence-based individualised nutrition interventions. Misinformation means the public and other healthcare professionals do not understand which interventions are effective and which are not; conflicting information means the public is confused and frustrated about what information is reliable. Continued efforts are needed to promote sources of accurate, consistent and reliable nutrition information. Dietitians and nutritionists are highly educated in this field and trained to understand and utilise credible sources, to critically evaluate information and to tailor information to the needs of the individual, so are an obvious source of high-quality information. Their influence is needed to improve the quality of media outputs so that the public have access to information that will help them optimise their health.

## Challenges and solutions

Several scientific and practical challenges are faced by those implementing research and guidelines for nutrition interventions in individuals. These include clear definitions and diagnostic criteria to enable the appropriate application of research and guidance; the application of population guidance to individuals, appropriate outcomes to allow monitoring and evaluation of treatment outcome; recognition of inter-individual variation and diversity of diet resulting in the need for highly tailored and individualised treatment approaches; and the need to translate evidence established on nutrients to advice based on the consumption of foods and the balance of the whole diet.

### Lack of consensus on definitions and diagnostic criteria

Without clear definitions of screening and diagnostic criteria, guidelines cannot be accurately applied to the individual. It is crucial to know which health conditions are amenable to dietary manipulation and under what circumstances. Diagnostic criteria must be robustly validated to identify the health condition in question and should be acceptable to the relevant population. An example where, on the surface, criteria seem clear are the WHO’s criteria for identifying obesity. The criteria use BMI^(86)^ based on an association with mortality. These provide a starting point for identifying health risk associated with excess body fat. However, the diagnostic sensitivity of BMI is reduced in well-muscled or very tall people^(87)^ and needs adaptation for different ethnicities^(88)^. Furthermore, the use of BMI as a sole diagnostic criterion for obesity has been contested in the 2020 Canadian Adult Obesity guideline, which recommends the use of BMI for screening purposes, but requires clinical indicators, such as waist circumference and evidence of cardiometabolic risk, for diagnosis^(89)^. Thus, although the criteria are widely used their application cannot be universal and professionals need to be aware of the exceptions and alternatives, for example waist to height ratio^(90,91)^. Similarly, criteria for identifying malnutrition have been challenging to develop. Numerous criteria have been proposed and trialled, but these have varied with population and setting. The Global Leadership Initiative on Malnutrition recently developed diagnostic criteria^(92)^ that provide an opportunity for a coherent and consistent approach universally. At this time and despite more than 79 studies, the validity of the Global Leadership Initiative on Malnutrition criteria is not established^(93)^. Currently, a major USA malnutrition validation study is in progress to help clinicians diagnose malnutrition (undernutrition) in adults and children^(94,95)^. The development of criteria requires good quality research that confirms its internal and external validity and, where possible, also evaluates the prognostic value in a wide range of settings; this is time-consuming and requires expertise in nutrition. No diagnostic criteria will have 100 % sensitivity and specificity in all populations and settings, but consistently applying evidence-based criteria and using clinical judgement will increase their utility. It will also facilitate the generation of evidence and its synthesis in meta-analyses where a variety of widely used criteria are accepted if a single clear definition does not exist.

### Applying population-based guidance on nutrient intakes to individuals

The quantity of each nutrient required by individuals is influenced by several factors including their age, sex, physiological and health status, body size and total food intake and this leads to potentially great variation in requirements between individuals. National and international guidance on reference nutrient intakes (RNI) has been developed but these focus on the needs of populations who are in good health rather than for individuals of different health status. For example, in the UK, the RNI for protein and micronutrients represent the amounts that are estimated to be sufficient for 97·5 % of a healthy population (Dept of health 1991). The RNI values are derived statistically from population requirement data and, for most nutrients, are equivalent to the mean population requirement plus two sd. When using RNI values to guide the intake of a given individual, it must be remembered that this will not be sufficient for 2·5 % of the population. However, without further individualised information, it is not possible to identify who will need more than the RNI. For energy, total fat and total carbohydrate intake, guidance is expressed only as estimated average requirement based on a mean healthy population requirement. estimated average requirement and not RNI values are provided because population intakes that would meet the needs of 97·5 % of the population would lead to obesity in many people^(96,97)^. It is, therefore, important to recognise that approximately 50 % of healthy individuals will require an intake above the estimated average requirement and 50 % will require less than the estimated average requirement and this leads to considerable uncertainty about the precise amounts an individual requires. In addition, for individuals who are not in good health or who are consuming an atypical intake (e.g. provided by a highly restricted range of foods or an extremely coarse diet very high in unrefined cereals where digestion and absorption may be reduced) application of population guidance on nutrient intake to meet their individual requirement is likely to be even more uncertain.

The key point is that guidance on intakes for healthy populations must be interpreted with caution when applied to an individual. Making such interpretations is a key competency of nutrition and dietetic professionals. Researchers will usually design nutrition interventions based on population guidance and thus, the results of these studies also need to be interpreted carefully in their application to individuals.

### Choosing the most appropriate outcomes

Clinical guidelines recommend the best treatment based on the evaluation of an intervention’s effect on specific outcomes. However, the complexity of some health conditions, the length of time since disease onset and the complexity of interventions mean that nutrition interventions are often evaluated using intermediate outcomes (biochemical markers, anthropometric measurements etc.) rather than clinical endpoints (mortality, disease remission etc.) or outcomes valued by the individual concerned (maintaining independence, healthy life expectancy etc.). The development of nutrition-specific patient-reported outcome measures has potential to focus research on what is most important to those receiving the intervention. The International Consortium on Health Outcomes Measurement works to define, standardise and implement outcome measures that really matter to patients, for example the tracking of dietary issues in colorectal cancer^(98)^. Patient-reported outcome measures have been predominantly developed to explore single interventions that can be delivered in a uniform way to large numbers of patients, for example ophthalmic and orthopaedic surgery^(99,100)^. In medical conditions requiring nutrition interventions, for example diabetes^(101)^ and inflammatory bowel disease^(102,103)^, treatment is often combined with other medical interventions, for example medication or physical activity, making it difficult to identify the effect on outcomes that are due to nutrition alone. In addition, there is evidence that patients’ preferred outcomes from nutrition interventions include wide-ranging domains, such as food intake, quality of life and functional ability. For example, in liver and coeliac disease it is difficult to attribute any benefits solely to changes in nutrition intake^(104,105)^. In a recent investigation of real-world evidence, it was shown that patient-reported outcome measures are not routinely reported and more work is needed in this area to raise awareness among those working in nutrition and dietetics about the importance of tracking patient-reported outcome measures as part of evidence-based nutrition care^(106)^.

Evaluating nutrition interventions is particularly challenging as the time frame of anticipated benefits is frequently long-term and beyond the usual limits of data collection. For example, nutrition interventions may impact on constipation within weeks, but their influence on hospital admission for diverticular disease may take longer to become apparent (Carabotti *et al.* 2021). Similarly, adherence to a Mediterranean diet (described as **i**ncluding abundant plant-based foods, provides olive oil as the primary source of fat, and includes low to moderate amounts of fish, meat, dairy products, eggs and wine) is associated with an observable reduction in total and LDL-cholesterol within weeks^(107)^, but it may take several years for a decrease in major cardiovascular events to be identified^(108)^. Many systematic reviews of nutrition interventions report limited conclusions due to the poor quality of the included studies^(109–111)^, thus hampering the production of clinical practice guidelines. Designing studies to examine the effects of nutrition interventions and to inform guidelines, requires the selection of appropriate outcomes that are relevant, measurable and specific to nutrition and that data are collected over sufficient durations.

### Applying evidence in the context of complex individual situations

A further challenge is the immense inter-individual variation in the context of interactions between health conditions and people’s real lives, which need to be considered when planning and delivering nutrition interventions. Approximately half of adults aged ≥ 65 years have three or more health conditions^(112)^ leading to complex treatment needs, which potentially include diverse or contradictory dietary needs. For example, a patient with diabetes and cardiovascular disease who develops chronic kidney disease will need each comorbidity to be considered separately as well as in combination with others and their diet adapted accordingly. If they go on to lose body weight and develop pressure ulcers, further dietary adaptation might be anticipated. Guidance on their nutrition intake should also take account of their personal food preferences and cultural practices, religious beliefs, or ethical principles as well as potential loss of appetite due to pain and/or depression. Overlaying financial, environmental, cultural, educational factors that impact food choice and the ability or facilities to prepare food adds yet more challenges^(1)^. This hypothetical complex example demonstrates the need to use the three elements of evidence-based practice; research evidence, clinical expertise and patient preference described earlier (Fig. 1(a))^(9)^.

It is unlikely that individuals with very complex needs would participate in most clinical studies evaluating nutrition interventions, as comorbidities are often explicit exclusion criteria, which raises the question of how to obtain evidence to inform dietary advice for these individuals. Reporting individual case studies^(113)^ or case series is potentially appealing in such situations but requires the author’s objectivity and readers’ caution in recognising the limitations and lack of generalisability of the evidence. Clinical guideline development processes also do not typically include this type of evidence, and invariably guidelines are not developed for people with multiple morbidities and highly complex needs. The professional is left to apply several guidelines for different conditions to one individual, which requires advanced levels of clinical skill, critical thinking, acumen and experience.

### The shift from nutrient research to food interventions

Providing nutrition interventions to individuals is largely articulated as *food*, including the types and combinations of foods to eat or avoid, the portions that are desirable and how these are prepared or cooked. This differs from recommendations about *nutrients*; for example, dietary reference values in the UK that are provided for groups of healthy people^(96)^. The translation from nutrients to food requires specialist knowledge of the nutrient/s which need adaptation for health, as well as knowledge and a full understanding of wider nutritional requirements. In addition, knowledge of micronutrient bioavailability from different food sources and interactions with other nutrients or with drugs may also be needed. These factors must then be adapted to the socio-economic and environmental situation, and cultural preferences of the individual, to communicate this effectively and to facilitate the required behaviour change^(114)^. A clinical guideline does not attempt to do this, and the professional’s skill is the application of rigorously collated high-quality evidence from a guideline to the highly individual circumstances of the patient. Nutrition and dietetic professionals learn this skill as part of their professional training and evidence-based practice and the application of research is a competency standard for nutrition and dietetic professionals as discussed previously. Additional research is needed to enhance professionals’ implementation of guidelines. Such research may focus on investigating measurement of guideline adoption and resulting outcomes^(73)^. This type of research can be instrumental to develop much-needed implementation tools and resources for nutrition and dietetic professionals.

### The involvement of patients and the public in the development of nutrition research

The design of nutrition interventions for individuals requires a clear understanding of patient’s values, preferences and experiences, yet there is very little research into this area. One solution is to involve patients and the public in the development of nutrition research to help ensure that research questions, intervention design and outcomes are relevant and meaningful to those who will ultimately benefit from the research. Patient and public involvement and engagement can also help ensure that research is patient-centred and that the research process is transparent and accountable. The most thorough processes for developing guidelines include phases where patients and other stakeholders input into the final document. Increasingly research funding bodies now explicitly require evidence of strong patient and public involvement and engagement; thus, the voice of the patient is starting to be heard.

In particular, involving the public can help ensure research findings are communicated in an understandable, accessible and transparent way and that guidelines are based on rigorous processes and high-quality research. Increasing public awareness of nutrition research and guideline development can also help highlight their importance in promoting health and preventing and treating disease, as well as increasing public understanding of the best sources of nutritional information. However, involving patients and the public in research has its challenges. There may be difficulties in identifying and engaging relevant stakeholders and ensuring that their perspectives are adequately represented. Additionally, it can be time-consuming and resource-intensive.

## Conclusion and Academy of Nutrition Sciences consensus recommendations

This paper has explained how nutrition research evidence is integrated into clinical practice and how it supports evidence-based practice for individual interventions. We have outlined specific challenges in the design and conduct of studies of nutrition interventions that can limit the certainty of the evidence and feasibility of carrying out some research. Guidelines support professionals delivering nutrition interventions to apply evidence appropriately and many organisations develop and publish such guidelines. The AND and PEN methods have been explained to illustrate different approaches to assisting professionals in applying research evidence when working with individual clients. Finally, we have highlighted some of the pressing challenges within the field of nutrition and dietetics in generating and implementing high-quality robust evidence for individualised nutrition interventions. The following consensus recommendations aim to offer potential solutions and improvements to these challenges.

### Consensus recommendations

The ANS makes ten consensus recommendations based on this paper, which are aimed at three different groups:

- those who deliver nutrition interventions to individuals (nutrition and dietetic professionals and their professional bodies who uphold standards)
- those funding, commissioning or undertaking research aimed at delivering evidence-based nutrition practice (e.g. grant funding bodies, guideline developers, researchers, etc.)
- those disseminating nutritional information to patients and the public (people in the media, journalists, policy-makers, politicians, other healthcare professionals, etc.)

#### Those who deliver nutrition interventions to individuals

1. An evidence-based approach to delivering nutrition interventions is crucial to ensure the intervention is efficacious and most likely to be acceptable, effective and safe. Nevertheless, it is important to recognise that the highest levels of evidence are sometimes not possible to achieve due to the nature of research in nutrition and diet, in humans. Therefore, the concept of using the entirety of the *best available* evidence should be applied in prescribing nutrition interventions in individuals by nutrition and dietetic professionals.
2. Nutrition and dietetic curriculums and competencies should be continually examined and reviewed to ensure nutrition and dietetic professionals are trained in the skills outlined in this paper:
  a. reviewing, critiquing and applying best available research evidence for nutrition interventions in individuals
  b. identifying where research evidence is lacking and having the skills to design and conduct research to fill these gaps
  c. understanding systematic reviewing and guideline development processes so these are undertaken routinely, and the best available evidence is applied to practice
3. Nutrition and dietetic professionals should be trained and maintain their competency to combine all relevant factors when advising clients in the context of the best available evidence (e.g. cultural, personal, medical, environmental, societal).
4. Leadership is required from professional bodies to acknowledge and pursue evidence-based practice. This includes raising awareness of high-quality dietary information, particularly with policy-makers and other stakeholders. Continued efforts are needed to promote nutrition and dietetic professionals as the best source of nutritional and dietary information and guidance, as they have the skills required to navigate the complexity of applying evidence to individualised care. Targeted outreach campaigns that aim to increase awareness and build trust could be important approaches that professional bodies may pursue systematically.

#### Those funding, commissioning or undertaking research

1. Patient and public involvement and engagement should be prioritised and included as part of funding criteria for future nutrition-related research. It is crucial in priority setting and research design to ensure patient values, preferences and experiences are incorporated.
2. Research is needed to understand the barriers and facilitators to guideline implementation, and priority should be given to this area. Now that defined systematic processes have been created to develop high-quality guidelines, work is needed to ensure that they can be implemented and applied in clinical practice to ensure patients receive care based on the best possible evidence.
3. A greater understanding of the most robust research designs for use in nutritional interventions aimed at individuals is required. The development of a hierarchy of evidence specifically for nutrition studies for individualised care is needed, which reflects the concepts of study quality, best available evidence and individualisation.
4. Understanding the extent of nutrition misinformation and identifying solutions to tackle it is a key research priority. We need to understand what sources of nutrition information people use, the quality of the information provided from different sources, and where the highest risk of misinformation lies. There is a need to help different groups (patients, public, policy-makers, other healthcare professionals etc) to distinguish between ‘good’ and ‘bad’ information and identify sources of high-quality information, such as the promotion of health information certification schemes^(115)^.

#### Those disseminating nutritional information

1. People conveying research findings or other nutritional information should acquire the skills to interpret scientific data (or work closely with professionals who have these skills) and identify sources of trusted reliable information. As this paper has detailed, nutrition and dietary advice to individuals is rarely simple. Those disseminating nutritional information need to ensure they are able to provide safe, useable, relevant dietary information to individuals.
2. Trusted and reliable sources of information for individualised advice include professionals who are suitably qualified, having the in-depth understanding of the evidence and the skills to critically evaluate it, and practice under a code of ethics, such as dietitians and nutritionists who are credentialled with their national authority or registration body. These professionals should be the preferred source of information.
